# Citrous Lime—A Functional Reductive Booster for Oil-Mediated Green Synthesis of Bioactive Silver Nanospheres for Healthcare Clothing Applications and Their Eco-Mapping with SDGs

**DOI:** 10.3390/molecules28062802

**Published:** 2023-03-20

**Authors:** Nasir Sarwar, Muhammad Shahzad Zafar, Usama Bin Humayoun, Suhyeon Kim, Syed Waqas Ahmad, Yong Ho Kim, Dae Ho Yoon

**Affiliations:** 1 Department of Textile Engineering, University of Engineering & Technology, Faisalabad Campus, Lahore 38000, Pakistan; 2 School of Advanced Materials Science and Engineering, Sungkyunkwan University, Suwon 16419, Republic of Korea; 3 Department of Chemical Polymer and Composite Engineering, University of Engineering & Technology, Faisalabad Campus, Lahore 38000, Pakistan; 4 SKKU Advanced Institute of Nanotechnology (SAINT), Sungkyunkwan University, Suwon 16419, Republic of Korea

**Keywords:** sustainable development goals, nontoxic, silver nanoparticles, medical textile, multifunctionality

## Abstract

Silver nanoparticles (Ag-NPs) are most effective against pathogens and have widely been studied as antibacterial agents in commodity clothing, medical textile, and other hygiene products. However, prolonged utilization of silver and rapid mutation in bacterium stains has made them resistant to conventional silver agents. On the other hand, strict compliance against excessive utilization of toxic reagents and the current sustainability drive is forcing material synthesis toward green routes with extended functionality. In this study, we proposed an unprecedented chemical-free green synthesis of bioactive Ag-NPs without the incorporation of any chemicals. Cinnamon essential oil (ECO) was used as a bio-reducing agent with and without the mediation of lime extract. A rapid reaction completion with better shape and size control was observed in the vicinity of lime extract when incorporated into the reaction medium. The interaction of natural metabolites and citrus compounds with nanoparticles was established using Fourier transform infrared spectroscopy (FTIR) and Raman spectroscopy. The application of as-prepared nanoparticles on textiles encompasses extended bioactivity to treated fabric with infused easy-care performance. To the best of our knowledge, this is the first reported instance of utilizing bioactive silver nanoparticles as a functional finish, both as an antimicrobial and as for easy care in the absolute absence of toxic chemicals. The easy-care performance of fabric treated with lime-mediated nanoparticles was found to be 141^O^, which is around 26% better than bare cotton without any significant loss in fabric strength. Furthermore, to enlighten the sustainability of the process, the development traits were mapped with the United Nations Sustainable Development Goals (SDGs), which show significant influence on SDGs 3, 8, 9, and 14. With the effective suspension of microorganisms, added functionality, and eco-mapping with SDGs with the chemical-free synthesis of nanoparticles, widespread utilization can be found in various healthcare and hygiene products along with the fulfillment of sustainability needs.

## 1. Introduction

Clothing material subjected to a fibrous structure and sufficient moisture retention provides a suitable environment for the proliferation of microbes. The rapid growth of microbes (fungi and bacteria) close to the skin (on clothing) causes a severe impact on human health by causing direct sickness in humans along with easy and rapid dispersal while moving. Bacterial infections alone are responsible for 20% of all fatalities in the world [1]. Such contaminated textiles, on the other hand, are a potential source of nosocomial infection transmission to other patients and medical or paramedical workers, particularly in the hospital environment. Silver, being the most effective against microbes, is widely used as an antibacterial and antifungal in textile finishing [2]. The rapid interaction of silver ions with the bacterial cell wall leads to structural distortion, along with slowing down of cell activities and eventually cell death [3,4,5,6]. A variety of silver-based finishes have been developed by researchers to restrict the development of bacteria; however, prolonged overuse of silver has hampered bacteria’s adaptability while rendering these finishes useless in the prevention of basic species [7]. On the other hand, the lack of bonding interaction of nanoparticles with substrate causes the easy release of nanoparticles while compromising on functional performance. The incorporation of various crosslinking agents (DMDHEU and DHEU) along with antimicrobial finish is often reported where these used chemicals have severe compliance-related issues along with the deterioration of fabric strength and aesthetic [8]. Further, the huge utilization of toxic chemicals and reagents in the synthesis of source silver material is another constraint. The current sustainability wave and ZDHC drive have applied strict legislation limits on the utilization of toxic reagents even in the synthesis of raw materials, especially for the textile and leather industry [9,10]. The increased awareness about personal health and hygiene, after COVID-19, has also increased the huge demand for green material synthesis and functional textiles with extended antimicrobial and antifungal properties. To this aspect, researchers have employed various nanoparticles (NPs), particularly silver-based NPs, to eliminate infections caused by bacteria [11,12] and fungi [13]. Ag-NPs possess distinctive characteristics, such as high surface area, stability, biocompatibility, and surface plasmon resonance, which render them highly fascinating for medical textile applications [14]. For the cleaner and sustainable production of silver nanoparticles, researchers have tried to avoid the traditional wet chemical process [15,16,17] and other microbial synthesis routes [18,19], being toxic and highly stereoselective, respectively. The reduction of metallic salt to metal NPs using plant extracts has been considered a low-cost, sustainable, and eco-friendly route, since plants contain proteins, sugars, flavonoids, alkaloids, steroids, and terpenoids, etc., that help in bio-reduction, stabilization, and capping in a single step and make the process simple and effective [20]. The synthesis of Ag-NPs has been widely explored using extracts taken from leaves, bark, stems, roots, and flowers [21]. Despite the potential benefits of using plant extracts to synthesize Ag-NPs in a sustainable manner, the incorporation of capping metabolites to enhance their properties has not yet been widely adopted in commercial applications due to associated shortcomings. Bio-reduction, for example, is a time-consuming process, and generating regularly shaped particles without aggregation remains a challenge [22,23]. Various external stimuli such as the utilization of plasma liquid [24], microwaves, or radio energy have been coupled in the green synthesis of nanomaterials; however, being subjected to high energy cost, these are not a sustainable solution [25,26,27]. The incorporation of various organic solvents and other chemicals has also been reported to boost the reduction process, but again with compliance-related issues [28,29]. Essential oils have the competitive edge over the plant extract being enriched with active agents and metabolites, but unfortunately, essential oils are less studied in green synthesis as compared to the corresponding extract. Matheus et al., in their recent work, synthesized Ag-NPs using a series of different natural oils (oregano, clove, rosemary, etc.) and evaluated the bioactivity against Gram-positive bacteria [30]. Ceylon Cinnamon (cinnamon) is known as a miracle plant due to a variety of health, medical, and cosmetic benefits. Being a strong antioxidant with a pleasant smell, it has been used in aromatherapy as a relaxant, detoxifying agent, stimulator for the limbic system, among many more uses [31,32,33,34]. Chemically, cinnamon is composed of eugenol, cinnamaldehyde, cinnamic acid, and a pool of different phenolic metabolites that impart strong antibacterial and antifungal properties. Nasir et al., used cinnamon bark extract along with citric acid mediation for the synthesis of copper nanospheres [35]. The utilization of cinnamon bark extract for the synthesis of Ag-NPs is also reported in the literature. Saliem et al., reported the synthesis of silver clusters by incorporation of cinnamon zeylanicum [36]. Premkumar et al., unfolded the green synthesis of metallic silver with the help of an aqueous solution of cinnamon powder. As-synthesized micro- to nano-sized particles were evaluated for bioactivity against various bacterial strains [37]. Jeevika et al., explore the seed-free synthesis of silver nanowires using clove oil [38].

Herein, we introduce a chemical-free green synthesis of biologically active Ag-NPs using cinnamon essential oil as a natural reducing agent. Taking advantage of the oxygen-scavenging ability and strong interaction of citric acid with metal nanoparticles, the lime extract was mediated in the synthesis process as a reduction booster cum capping agent. The as-synthesized NPs were conjugated onto the cotton fabrics and were evaluated for bioactivity against common human-disease-causing bacterial and fungus strains. The fabric tear strength and easy-care properties were assessed for the evaluation of functional performance. To the best of our knowledge, this is the first reported work showing the rapid and chemical-free synthesis of a regularly shaped nanosphere using cinnamon oil with effective bioactivity and extended functional performance in textiles. With a broad spectrum of antiseptic properties in chemical-free synthesis, such a material can find tremendous utilization in medical textiles, hospital bedding, and other healthcare and hygiene products by providing the next level of protection along with extended performance.

## 2. Results and Discussion

**Figure 1:**
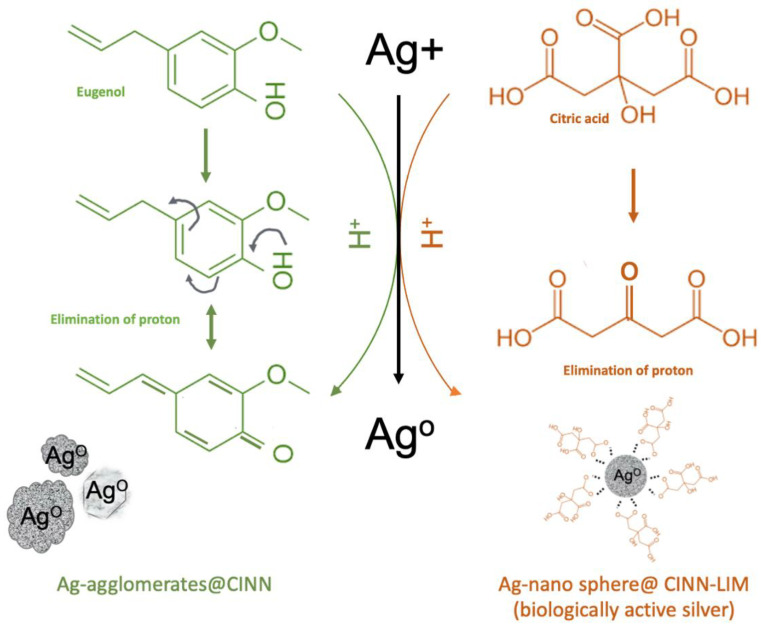
Possible reduction mechanism of silver in the presence of cinnamon and lime extract followed by capping.

Cinnamon essential oil is enriched with eugenol, which is a phenolic hydroxyl compound with potential reductive power [31]. As soon as Ag+ comes into contact with cinnamon oil, Ag^+^, due to its ability to accept a lone pair electron (due to empty s, p, and d orbitals of 3d^10^, S^o^ electronic configuration), is radially absorbed on eugenol to form a microemulsion [30]. The strong potential difference between eugenol (∼−1.5 v) and silver (∼0.8 V) results in the reduction of Ag^+^ to Ag^o^ (Figure 1) [35], which was observed with a gradual color change in the reaction medium within 8 h. In the presence of lime extract in the reaction medium, a rapid color change from yellowish to grayish was observed in 30 min. Lime juice is a rich source of citric acid—a weak organic acid with a strong ability to scavenge oxygen from the system [35]. The citric acid present in the reaction system performs a dual action, i.e., it synergistically promotes quick reduction along with size and shape control (Figure 1). As soon as Ag^+^ is reduced to Ag^o^, citrate ions are radially absorbed over the silver collide surface, capping the particles through the formation of a [Ag-citrate]^−^ complex, consequently preventing growth and size control and contributing to additional stability and functionality [15].

### 2.1. UV Adsorption Spectroscopy, SEM, and EDX Measurements

**Figure 2:**
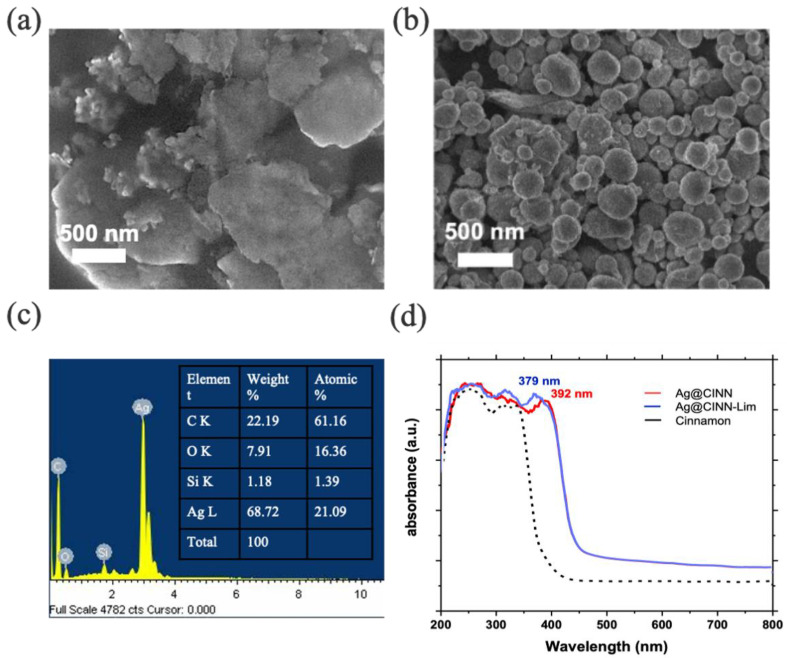
(**a**,**b**) SEM image of Ag@CINN and Ag@CINN-Lim nanoparticles. (**c**) The EXD spectrum (Ag@CINN-Lim). (**d**) UV–Vis spectrum of the source cinnamon and as-prepared nanoparticles.

A unique surface plasmonic resonance of nanoparticles in the UV absorption spectrum provides strong evidence of their formation [30]. The cinnamon oil shows absorption below the visible region in the range of 325–242 nm due to the presence of secondary metabolites, phonic compounds, and other benzene-based conjugate systems [37]. The as-prepared Ag@CINN nanoparticles indicate plasmonic absorption at 392 nm. Usually, Ag-NPs indicate an absorption peak in the visible region near 400 nm. In our case, the absorption spectrum of Ag-NPs was observed below 400 nm. This may be due to the effect of capping caused by secondary metabolites present in the cinnamon oil (Figure 2d) [37]. A blue shift in the UV–Vis absorption spectrum with a relatively higher peak intensity is referred to as the reduction in particle size and regular absorption from the surface of spherical-shaped nanoparticles (Figure 2d; blue line), as in the case of Ag@CINN-Lim [38]. SEM images provide a clear insight of the particle morphology and particle size in support of the UV spectrum. The Ag@CINN particles show an irregular shape that is potentially agglomerated together (Figure 2a). The lime-mediated Ag-NPs (Ag@CINN-Lim) present uniformly spherical shapes (Figure 2b). The radial absorption of the citrate compound over the nanoparticles directs the particle shape into a round sphere while citrate–citrate ionic repulsion reduces the agglomeration with better suspension [15]. The highest silver peak at 3 KeV in the EDX spectrum presents the strong existence of silver, whereas other existing elements in the spectrum might be attributed to the background and interaction of secondary metabolites and citrus compounds with nanoparticles (Figure 2c).

### 2.2. FTIR Spectroscopic Characterization

**Figure 3:**
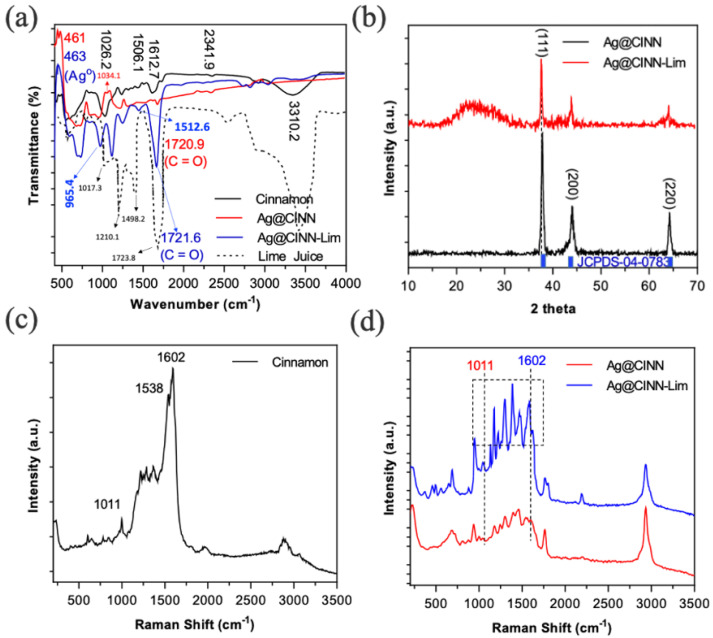
(**a**) FTIR spectra showing the interaction of metabolites with as-prepared nanoparticles. (**b**) XRD spectra showing diffraction peaks at 2 theta. (**c**) The Raman spectra of pure cinnamon. The spectra at 1602, 1538, and 1011 cm^−1^ correspond to -C=C- and C-C stretching and ether group deformation, confirming the eugenol and secondary metabolites present in cinnamon. (**d**) The Raman spectra of as-prepared samples. The elongation of peaks at 1011 and 1602 cm^−1^ is due to the capping of metabolites. The box represents the section in which the elongation of peaks is occurring in the vicinity of lime.

The FTIR spectra of as-prepared NPs in comparison to cinnamon oil and fresh lime juice also confirm the capping of secondary metabolites as well as citrus compounds over the nanoparticles (Figure 3a). Cinnamon essential oil is enriched with eugenol and cinnamaldehyde along with a sufficient number of secondary metabolites, cinnamaldehyde, phenols, and cinnamic acid. The peaks at 1612.7, 1506.1, and 1026.2 cm^−1^ (in the black spectrum) are the characteristic peaks of eugenol corresponding to benzene stretching vibration, C-H deformation, and aromatic ether vibration of O-C-O groups, respectively [38]. A wide dip between 3100 and 3600 cm^−1^ (in the black spectrum) is attributed to the -OH vibrational mode [13]. The appearance of the weak signature at 1034.1 cm^−1^ is attributed to the interaction of eugenol (present in cinnamon oil) with silver nanoparticles to a limited extent. The peak at 1720.9 cm^−1^ in the same spectrum (red line) might be attributed to the -C=O group of cinnamaldehyde—another active component in cinnamon oil [18]. The absorption peaks at 1017.3 and 1210.1 cm^−1^ in lime extract (dotted spectrum) are indicative of polyols (flavones, etc.) and N-O stretching, respectively. The sharpening of corresponding eugenol peaks (1512.6 and 965.4 cm^−1^) in the spectrum of Ag@CINN-Lim is indicative of the strong absorption capacity of these cinnamon functional groups in the vicinity of citrus lime, whereas a very sharp signature at 1721.6 cm^−1^ is due to the strong affinity of citric acid for metallic nanoparticles [16] while making them biologically more active [35].

### 2.3. The X-ray Diffraction (XRD) Characterization

The XRD pattern provides deep insight into the crystal structure and phase of the materials. Figure 3b provides the XRD spectra of the as-prepared Ag@CINN and Ag@CINN-Lim. The appearance of characteristic peaks with 2 theta diffraction values at 37.92°, 44.31°, and 64.47° align with the standard JCPDS # 04-0783 of bulk silver, unraveling the formation of pure metallic Ag nanoparticles in both cases without any corresponding peak of AgO [21], whereas these diffraction peaks correspond to (111), (200), and (220) phases of the FCC crystal structure of Ag-NPs. The synthesis of metallic silver was validated by calculating the lattice constant and d-spacing (Table S1). A broad peak appeared in the sample (Ag@CINN-Lim) (where lime was incorporated in the experimental setup), which is the disruption in the crystal structure that arises as a result of the strong interaction of capping metabolites and other compounds with nanoparticles [39].

### 2.4. Raman Spectra Characterization

The Raman spectroscopy was performed at 532 nm wavelength to further confirm the properties of Ag-NPs upon capping with lime, which was compared with other samples. The -C=C-, C-C stretching and ether group deformation peaks at 1602, 1538, and 1011 cm^−1^ confirm the presence of eugenol and secondary metabolites in cinnamon [40]. Moreover, the appearance of corresponding peaks of cinnamon in as-prepared nanoparticles with varying intensities in Ag@CINN and Ag@CINN-Lim can be seen (Figure 3c,d). Relative higher peak intensity presents better capping in Ag@CINN-Lim.

### 2.5. The Anti-Bacterial Activity Testing

**Figure 4:**
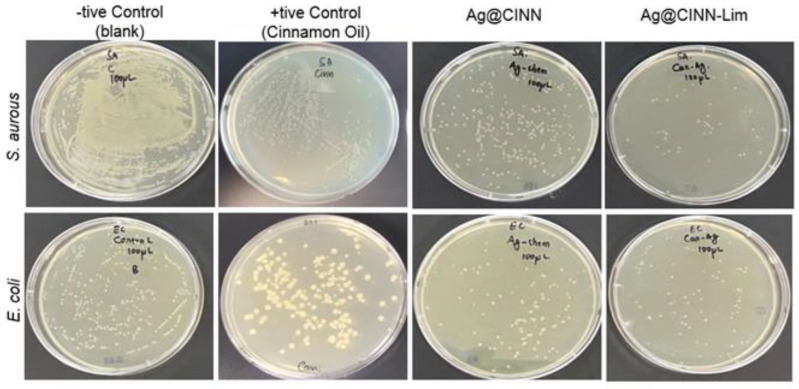
Photographic image of agar plate showing the intensity of bacterial colonies grown against each test.

As previously stated, Ag is well known for its antimicrobial properties and pathogenic protection and has maintained a sound position in the field of medicine, healthcare, sanitary products, and medical textile application since ancient times. However, the excessive utilization of toxic chemicals in the synthesis of Ag-NPs and the current wave of personal care and healthcare after COVID-19 have created a huge demand for green synthesized bioactive antimicrobial agents. In the above scenario, we evaluated our as-prepared bioactive Ag@CINN-Lim nanoparticles against Ag@CINN nanoparticles for antibacterial and antifungal protection. A series dilution was prepared to make the grown colonies countable, and % reduction was calculated compared to the blank sample plate. Figure 4 presents the photographic images of the incubated agar plates of all test samples along with the control, and the details of the samples along with respective colonial counts are shown in Table 1.

**Figure 5:**
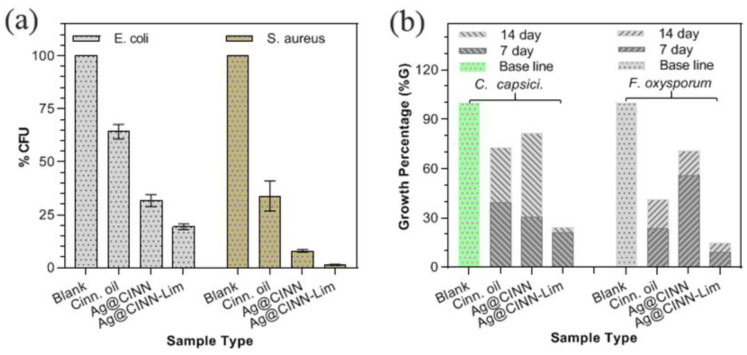
The growth percentage of our tested samples Ag@CINN and Ag@CIMM-Lim with controls (**a**) against bacteria (**b**) against fungi.

All of the experimental investigations were triplicated, as aforementioned, to eliminate experimental error; the results can be seen in Table S2. Based on the colonial count value, calculated by Equation (1), the resulting bar graph is presented in Figure 5a. The graph presents the average value of three tests, and the error bars represent the standard deviation, while the detailed photographic images of the agar plate for each repeated test are shown in Figure S1.

Aggressive colonial growth of the *S. aureus* and *E. coli* bacteria was found on the blank plate. It can be seen that the number of colonies on the blank plate with *S. aureus* was un-countable. To estimate the exact number of colonies, we diluted the bacterial medium in PBS buffer to various dilution folds and inoculated it on separate plates to count the actual number of colonies (Supplementary Material). The bacterium collected from the glass vail containing cinnamon oil-treated fabric showed relatively higher bacterial growth (65% for *E. coli* and 34% for *S. aureus*) on the agar plate as compared to Ag@CINN (32% for *E. coli* and 8% for *S. aureus*) and Ag@CINN-Lim (19% for *E. coli* and 1% for *S. aureus*). Cinnamon is well known for its antiseptic properties. The cinnamaldehyde, eugenol, and active metabolites present in the cinnamon oil inhibit bacterial growth by damaging the bacterial cell wall through modification of the lipid profile of the bacterium cell wall. Ag@CINN and Ag@CINN-Lim showed effective inhibition against both Gram-positive and Gram-negative bacteria. In the case of Ag@CINN, diffusion of Ag-NPs in the damaged cell wall change the cell wall structure via interactions with sulfur proteins, which slow bacterial metabolism and consequently cause cell death. Lime-mediated silver nanoparticles (Ag@CINN-Lim) were found to be much more effective as compared to bare nanoparticles prepared using cinnamon oil only (i.e., Ag@CINN). Citrus extracts are enriched with citric acid with an intrinsic ability to inhibit pathogen growth. Microbes are stereoselective toward pH [11]. The adsorption of citrus compounds (present over the nanoparticles) contributes to the rapid bacterial suspension due to its strong disruptive effect on the cell membrane. Once Ag@CINN-Lim is adsorbed inside the cell membrane, the -COOH anion leads to the acidification of the cytoplasm of the bacterial membrane, causing rapid damage and extended suspension [5,35].

### 2.6. The Anti-Fungal Activity Testing

**Figure 6:**
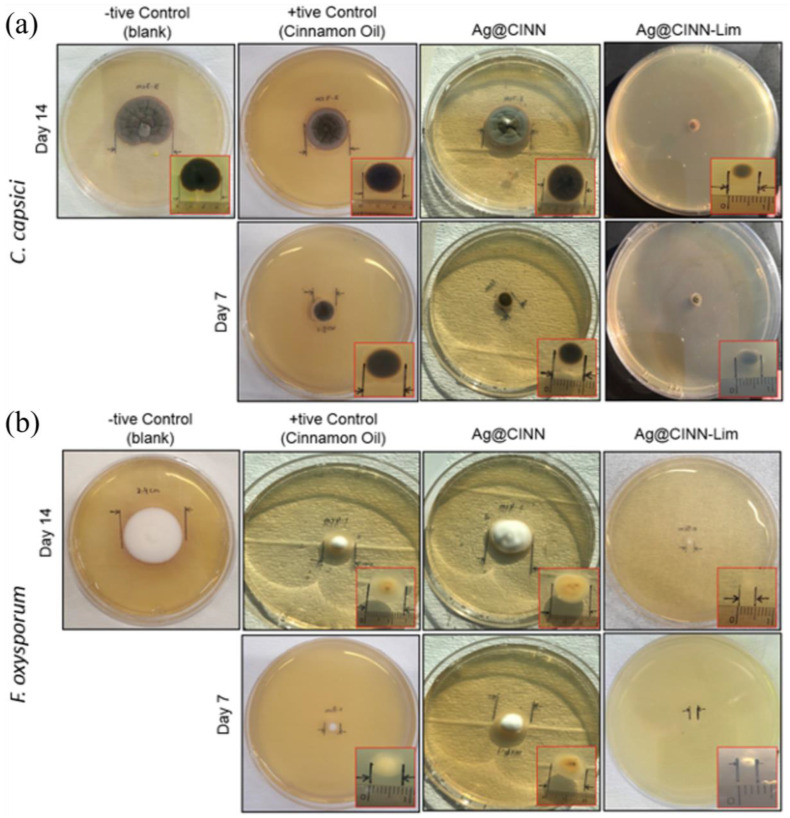
Photographic image of agar plate showing the growth of fungi: (**a**) *C. Capsici* (**b**) *F. oxysporum*.

Inspired by the previous studies of employing botanical extracts against fungi, we tested our prepared NPs for their antifungal activity. The results of the antifungal potential of our Ag@CINN-Lim are presented in Figure 6, along with positive and negative control samples. The plates positioned by our cinnamon-based Ag-NPs inhibit the mycelial growth of test fungi when compared with the control plates (without NPs: negative control; cinnamon oil: positive control). The diameter of the mycelial growth was noted after 7 and 14 days of growth under controlled conditions. The growth of *C. Capsici* after 14 days was 3.3 cm in diameter, as shown in Figure 6a. With this as the baseline, the percentage of mycelial growth (%G) was calculated by Equation (2), and the data are presented in Table S3.

The plate containing cinnamon oil (positive control) as the inhabitant showed 39.39%G after 7 days and 33.31%G in the next 7 days, with a total of 72.7%G after 14 days, as shown in Figure 5b. In comparison, the plate containing Ag@CINN showed relatively large percentages of growth, i.e., 30%G in 7 days and a cumulative of 81.82%G in 14 days. When subjected to cinnamon essential oils, their intrinsic ability first affects the cell membrane and attenuates efflux pumping, consequently inhibiting fungal growth [18]. The plate poisoned with Ag@CINN-Lim nanoparticles showed a very small percentage of growth as compared to the other samples, i.e., 24.2%G. The improved antifungal action of Ag@CINN-Lim might be due to the combined effect of cinnamon and citrus capping. The citric acid in citrus extract stops the nutrient supply to microorganisms by preventing pseudohyphae formation in yeast [41]. Hence, Ag@CINN-Lim well prohibited the growth of *C. capsici*. Similarly, growth of *F. oxysporum* (3.4 cm) was noted, as shown in Figure 6b, and was set as a baseline to estimate the growth percentage. It can be seen that the cinnamon oil allowed for 23.5%G in the 1st week and 17.7%G in the 2nd week, with a cumulative total of 41.2%G. In comparison with both the fungi strains, the cinnamon oil showed better efficiency against *F. oxysporum* by suppressing 31.5% of additional growth.

Further, Ag@CINN exhibited poor efficiency in suppressing growth, i.e., a cumulative growth of 70.6%G. However, when we prepared the Ag-NPs by our novel route (Ag@CINN-Lim), it showed a minute %G after the 1st and 2nd weeks, i.e., 8.8 and 5.9%G (sum of 14.7%G). The reduction % trend is expressed in Figure 5. In conclusion, our as-synthesized Ag@CINN-Lim NPs were found to be much more effective and biologically active than Ag@CINN with a 75.8% suspension of *C. capsici* and 85.3% of *F. oxysporum* in the 14 days of testing. The drawn comparison of our work to other similar reported works (using cinnamon in the synthesis of Ag nanoparticles) further elaborates the superiority of our adopted synthesis route (Table 2).

### 2.7. Assessment of Fabric Functional Performance and Eco-Mapping

**Figure 7:**
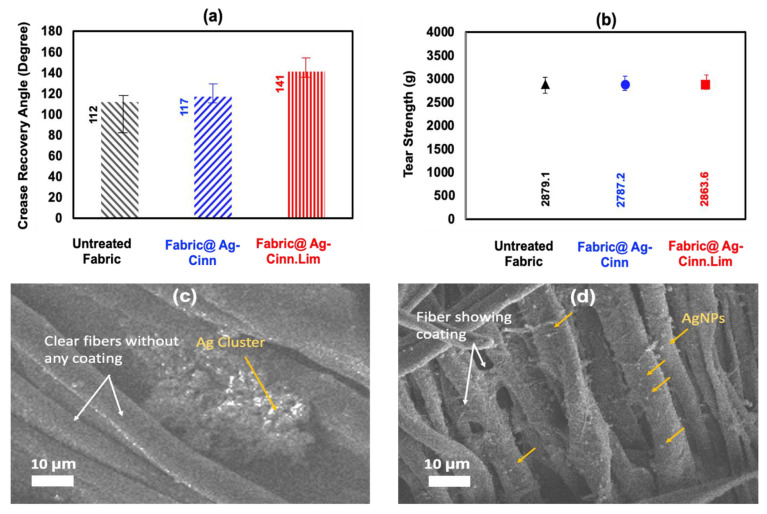
Measurement of fabric easy-care performance and tear strength as an impedance of crosslinking: (**a**) comparison of crease recovery angle value; (**b**) comparison of crease recovery angle value; (**c**,**d**) SEM images of fabric treated with Ag@CINN and Ag@CINN-Lim, respectively (yellow arrows point out the nanoparticles whereas white arrows point out the textile fibers).

**Figure 8:**
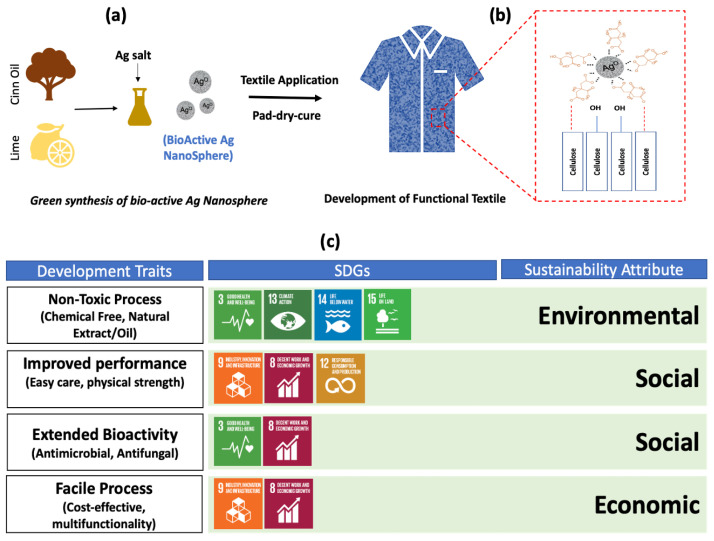
Utilization of bioactive silver nanoparticles for a sustainable functional textile: (**a**,**b**) development of functional textile with crosslinking mechanism; (**c**) eco-mapping of development traits with SDGs and sustainability attributes.

The rationale for testing the fabric easy-care performance is the ability of the citric compound to develop crosslinking with cellulosic materials due to the citric capping agent. In cotton fabric, the hydrogen bonding holds the cellulose chains together. The shift in these H-bonds to a new place under stress results in severe creasing in the cellulosic fabric. The crosslinking of the citric compound with the cellulose resists the shifting of bonding to a new place, consequently, an improvement in the crease recovery angle [44]. The capping of citric acid over the as-prepared nanoparticles has a fair chance to develop crosslinking with the fabric and the improvement in fabric easy-care performance [45]. To validate the claim, both fabrics treated with Ag@CINN and Ag@CINN-Lim nanoparticles were evaluated for easy-care performance against the untreated fabric. For ease of identification, the sample treated with Ag-CINN nanoparticles is referred to as F1 while that treated with Ag@CINN-Lim is referred to as F2 here onward. The untreated fabric presents a crease recovery angle value of 112°, while for samples F1 and F2, the crease recovery angle values were found to be 121° and 142°, respectively (Figure 7). The noticeable increase of 30° in the CRA value of sample F2 indicates the ability of citric-capped nanoparticles to crosslink with the cellulosic fabric [44]. A negligible increase of 9° in the crease recovery angle value was observed in the case of sample F1, where the fabric was treated with Ag@CINN nanoparticles. This slight increase in the CRA value of sample F1 might be subjected to the ability of cinnamaldehyde (present in cinnamon oil) to develop crosslinking. The physical incidences of crosslinking in the SEM images (Figure 7c,d) of samples F1 and F2 also seem to be in line with the depicted results, while the crosslinking mechanism is ascribed in Figure 8b.

A reduction in fabric strength upon crosslinking is commonly observed. In our case, no significant change in fabric strength of treated fabric was observed. Despite the improvement in CRA value, sample F2 still retained significant strength. The penetration of spherically round nanoparticles in between fiber interspaces increases inter-fiber cohesion, which enabled sample F2 to compensate for strength loss caused by esterification [45]. The textile and leather industry is under the keen observation of international policymakers, being the most influential one concerning environmental pollution. Material technologists and fabric scientists all over the world are continuously trying their best to develop a clean textile process with added performance and extended bioactivity, especially after the breakout of COVID-19. Although scientists have reported different aspects to improve the healthcare and hygiene of textiles, the eco-mapping of development traits with SDGs and sustainability is still lacking. Figure 8c provides the blueprint to interlink the development traits with sustainability building blocks. The green synthesis of bioactive silver nanoparticles and their utilization as functional finishing for the development of functional antimicrobial textiles sway the environmental and social aspect of sustainability with a direct impact on Sustainable Development Goals (SDGs) 3, 13, 14, and 15. The utilization of bioactive clothing developed through a non-toxic process with zero chemical addition directly influences good health and wellbeing, both for the manufacturer and consumer, whereas a harmless process drain prevents water pollution and soil erosion. Strength is the key performance indicator for durability, while batter crease recovery angle value contributes to better serviceability and aesthetic performance along with saving ironing costs. Our as-developed process incorporates multiple functionalities to processed textiles in the absolute absence of chemicals along with making the fabric biologically active against germs, which directly correlate SDGs 8, 9, and 12 (Figure 8c).

## 3. Material and Methods

### 3.1. Materials

Silver nitrate (99.9% pure) was purchased from Sigma Aldrich, Seoul, Republic of Korea. The cinnamon essential oil was sourced from Avi Naturals India (New Delhi, India). For citrus extract, lemons were purchased from the local market, and freshly squeezed juice was used. Tween 80 (CP grade) was purchased from Daejung Chemical (Siheung, Republic of Korea). All the glassware was thoroughly washed with deionized water and UV dried before use.

### 3.2. Methods

#### 3.2.1. Preparation of Ag-NPs

**Figure 9:**
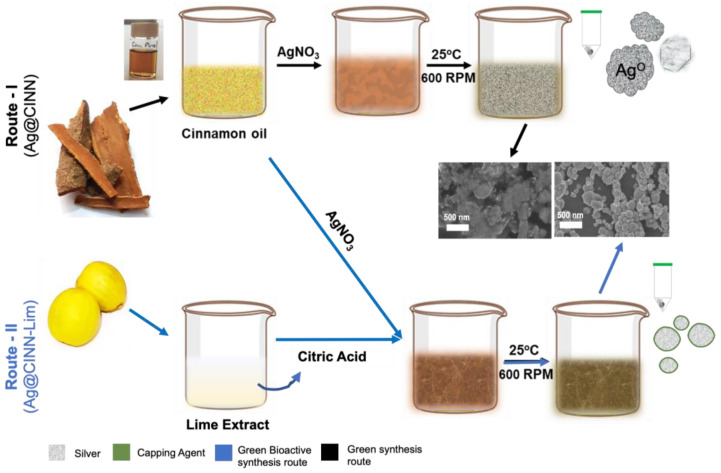
Schematic presentation of green synthesis of bioactive silver nanoparticles.

For the green chemical-free synthesis of Ag-NPs, cinnamon essential oil was used as a natural reducing agent, while lime extract was incorporated in the synthesis recipe as a reduction booster and capping agent. For typical synthesis, 10 mL of cinnamon essential oil (CEO) was taken in a glass vial, and the temperature of the experimental setup was increased to 60 °C while keeping it on the hot plate. Then, 50 mM (85 milligrams) of solid AgNO_3_ was poured into the vial containing CEO at a temperature of 60 °C and was kept stirring at 600 RPM. The synthesis of Ag-NPs was observed with the change in color with time spam. The yellowish-gray colloids of as-synthesized nanoparticles (after 8 h) were centrifuged at a speed of 10,000 RPM for 10 min while supernatant oil was restored for reuse. For the synthesis of biologically active green Ag@CINN/LIME, 1 mL of fresh lime juice (Lisbon Lemon) was added to the same recipe along with 0.1 mL of tween-80 as an emulsifying agent. A quick color change was observed in the case where the lime extract was added. After the removal of supernatant oil, the as-prepared nanoparticles were thoroughly washed with acetone and DI water each after the other, followed by vacuum drying. After drying, particles were redispersed in isopropyl alcohol and stored at room temperature for use. The schematic presentation for the synthesis process of biologically active Ag-NPs is described in Figure 9.

#### 3.2.2. Characterizations of Ag-NPs

The plasmonic resonance spectrum of as-prepared nanoparticles was recorded using NIR–UV visible spectrophotometer (model: Cary-7000 Agilent Technologies, Santa Clara, CA, USA) in a scan range of 200–800 nm. Surface morphology of the as-prepared nanoparticles was observed using a scanning electron microscope (model: FE.SEM-JEOL.7500-F, Gyeonggi-do, Republic of Korea), while phase identification of nanocrystals was made through X-ray diffraction (model: D8-FOCUS; Bruker, Billerica, MA, USA) in a scan range of 10–70° operated at a speed of 2s/step. Fourier transform infrared spectrophotometer of Nicolet (model: iD5-ATR) (Thermo Fisher Scientific, Waltham, MA USA) and Raman (WITEC, Ulm, Germany, ModelALPHA300M) were used for the identification of functional groups in cinnamon source and as-prepared nanoparticles.

### 3.3. Antimicrobial Assessment

The antimicrobial activity of as-prepared Ag-NPs was assessed against *Escherichia Coli* (Gram-negative) and *Staphylococcus aureus* (Gram-positive) bacterium as an antibacterial agent. Being highly resistive and common infection-causing strains, *E. coli* and *S. aureus* were chosen for antimicrobial performance as benchmarking stains. A well-known colonies forming unit (CFU) count method was adopted for the evaluation of antibacterial performance. For the adaptation of Ag-NPs as the textile finish, the Ag-NPs solution of concentration 1 mg/mL was prepared in water as adopted in the literature [35]. A piece of fabric was first padded with Ag-NPs solution at a wet pick up of 80% using padding mangle (Model: P-AO, HTAI, Jiangsu, China). After padding, the fabric piece was dried out in the oven at a temperature 100 °C followed by curing in a stenter (TD-600, Testex, Guangdong, China) at 170 °C for 2 min. For the anti-bacterial activity test, six glass vials were prepared separately, and then, treated fabric samples of size 1 × 1 cm were placed inside each vial. A 100 µL of separately grown unified bacterium inoculum of OD_600_ = 0.3 was then transferred to each vial containing treated and control fabric sample swatches. The bacterium test cultures were then allowed to be grown (overnight) on treated fabric inside the vial bottle, as the porous structure of the textile fiber provides a suitable surface for bacterial growth. Further, it provides a true comparison of utilizing the as-prepared nanoparticles for antimicrobial finish for healthcare and medical textile applications. After 8 h, bacteria grown over fabric (in the vial) were suspended in 5 mL of 1× phosphate buffer saline and diluted 10-fold followed by shaking well on an orbital shaker. The diluted solution (100 µL) from each vial bottle was drop cast onto TS agar plates, spread by a disposable plastic spreader, and the colonies were regrown overnight by placing them in an oven at 37 °C. The number of colonies grown on each TS agar plate was counted, and the % age count of colony growth was calculated using the control samples via Equation (1). The results were triplicated to reduce the possible error in the experiment.

Percentage count of colony growth (% CFU) = 100 − [(A − B/A) × 100](1)

where A and B are colonial counts in the reference plate and treated plate, respectively. The detailed method for preparation of TS agar plates for anti-bacterial assessment is explained in detail in Supplementary Material.

### 3.4. Antifungal Assessment

The poisoned food technique was conducted to evaluate the antifungal activity of as-prepared Ag-NPs, while a blank agar plate and plate with cinnamon essential oil (Raw) were used as negative and positive controls, respectively. Fungi, namely *Fusarium oxysporum* (from rhizome rot of ginger) and *Colletotrichum capsici* (from anthracnose of chili), being the most common, were employed to test bioactivity. The method for preparation of agar plates for anti-fungal activity testing is explained in detail in Supplementary Material. In brief, well-sporulated cultures of test fungi were inoculated on -tive control (blank without sample) and poisoned plates (with samples and simple cinnamon oil) by point inoculation using a sterile inoculation needle. The plates were incubated in an upright position for 14 days at room temperature. The diameter of colonies was measured in mutually perpendicular directions. The antifungal activity of NPs and control material, in terms of the percentage of mycelial growth (%) of test fungi, was determined using Equation (2).

Percentage of mycelial growth (% G) = 100 − [(C–T/C) × 100](2)

where C and T refer to the diameter of fungal colonies on control and poisoned plates, respectively.

### 3.5. Crease Recovery and Tear Strength Assessment

For the assessment of fabric easy-care performance standard BS-EN-22313 (1992), the method was adopted as per typical industrial norms. The fabric tear strength was assessed using ASTM standard method number D-1424. All samples were preconditioned at 20 ± 2 °C and 65 ± 4% humidity level for 24 h before performing any test.

## 4. Conclusions

In this study, shape-controlled bioactive silver nanoparticles were synthesized through a green synthesis process for functional medical textile applications. The process employed cinnamon essential oil as a natural reducing agent and lime extract as a reduction booster and functionalizing agent. The lime extract not only acts as a reactive booster to reduce the reaction time from several hours to 30 min, but it also caps the silver nanoparticles. The FTIR and Raman spectra confirmed the effective functionalization of the as-prepared silver nanoparticles with cinnamon secondary metabolites in the presence of citrus lime, while SEM images validated the better shape control. The as-treated fabric with lime-mediated silver nanoparticles presented remarked antimicrobial performance alongside the sufficient improvement of 30° in fabric easy-care performance without affecting fabric strength. The crosslinking mechanism of nanoparticles with cotton fabric was also drawn to physical conjunction in support of interlinking while other functional attributes were mapped, with sustainability showing a direct impact on SDGs 3, 8, 9 and 14. With drawn eco-mapping of SDGs with sustainability, this study provides new understanding of the chemical-free green synthesis of silver nanoparticles for functional textile applications without any toxic mediation.

## Figures and Tables

**Table 1:** Sample descriptions used for antimicrobial testing with respective colonial growth.

Sample Name	Description	Colonial Count	
		*S. aureus*	*E. coli*
−tive control	Blank without any treatment	3350	350
+tive control	Fabric treated with cinnamon oil	890	211
Ag@CINN	Fabric treated with Ag@CINN	248	119
Ag@CINN-Lim	Fabric treated with Ag@CINN-Lim	38	66

**Table 2:** Comparison of current research work with other related literature.

Source	Medication	Reaction Time		Reference
Cinnamon bark aqueous extract	No	8 h	Long reaction time Nanoclusters Bioactivity not measured	[36]
Cinnamon bark aqueous extract	No	2 days	Long reaction time Bioactivity against fungi is not measured	[37]
Cinnamon bark aqueous extract	No	8 h	Long reaction time Bioactivity not measured	[42]
Cinnamon bark aqueous extract	Microwave	1 day + microwave irradiation	Long reaction time High energy microwave utilization Bioactivity not measured	[43]
Clove oil	Acetone, NaOH	100 °C at 30 min	Bioactivity against fungi is not measured	[30]
Cinnamon oil	Lime extract	Room temperature at 30 min	Quick reaction completion Bioactivity against both bacterial and fungi	Our work

## Data Availability

Available on demand.

## Supplementary Materials

The following supporting information can be downloaded at: https://www.mdpi.com/article/10.3390/molecules28062802/s1, Table S1. Calculation of lattice constant and d-spacing for as-prepared Ag@CINN-Lim NPs; Table S2. The total number of colonies in all experimental runs; Table S3. Fungal activity triplicate data for Ag@CINN and Ag@CIMM-Lim with controls. Figure S1. Photographic images of incubated agar plates for each of the three repetitions; Figure S2. Estimated number of colonies in S. aureus (−tive control).
