# Supplementary material for: Citrous Lime—A Functional Reductive Booster for Oil-Mediated Green Synthesis of Bioactive Silver Nanospheres for Healthcare Clothing Applications and Their Eco-Mapping with SDGs
Source: Molecules. 2023 Mar 20;28(6):2802. doi: 10.3390/molecules28062802 (PMC10052960; doi:10.3390/molecules28062802)
Supplement: Supplementary file 1 [file molecules-28-02802-s001.zip › molecules-2228864-supplementary.pdf]

Supplementary Materials

Citrous Lime—A Functional Reductive Booster for Oil-Mediated Green Synthesis of Bioactive Silver Nanospheres for Healthcare Clothing Applications and their Eco-Mapping with SDGs

Nasir Sarwar [1,2,†], Muhammad Shahzad Zafar [3,4,†], Usama Bin Humayoun [1], Suhyeon Kim [4], Syed Waqas Ahmad [3], Yong Ho Kim [4] and Dae Ho Yoon [2,4,*]

[1] Department of Textile Engineering, University of Engineering & Technology (Faisalabad Campus), Lahore 38000, Pakistan
[2] School of Advanced Materials Science and Engineering, Sungkyunkwan University, Suwon 16419, Republic of Korea
[3] Department of Chemical Engineering, University of Engineering & Technology (Faisalabad Campus), Lahore 38000, Pakistan;
[4] SKKU Advanced Institute of Nanotechnology (SAINT) Sungkyunkwan University, Suwon 16419, Republic of Korea
* Correspondence: dhyoon@skku.edu; Tel.: +82-31-290-7388; Fax: +82-31-290-7371
† These authors contributed equally to this work.

Table S1. Calculation of lattice constant and d-spacing for as-prepared Ag@CINN-Lim NPs.

<table>
<tr><th rowspan="3">λ (Å)</th><th colspan="3" rowspan="2">Miller indices</th><th colspan="2" rowspan="2">Bragg's angle</th><th colspan="2">Experimental</th><th colspan="2">Standard</th></tr>
<tr><th>d-spacing (Å)</th><th>Lattice constant (Å)</th><th>d-spacing (Å)</th><th>Lattice constant (Å)</th></tr>
<tr><th>h</th><th>k</th><th>l</th><th>2θ</th><th>θ</th><th>$d_{hkl}=\lambda/(2\sin\theta)$</th><th>$d_{hkl}\sqrt{h^2+k^2+l^2}$</th><th colspan="2">JCPD# 04-0783</th></tr>
<tr><td>1.5406</td><td>1</td><td>1</td><td>1</td><td>37.92</td><td>18.96</td><td>2.3703</td><td>4.1056</td><td>2.3590</td><td>4.09</td></tr>
</table>

Section I: Material Preparation for Antibacterial Activity (TS Agar Plate)

The antimicrobial activity of silver nanoparticles (Ag@CINN and Ag@CINN-Lim) was assessed against *Escherichia Coli* (Gram-negative) and *Staphylococcus aureus* (Gram-positive) bacterium via colonies forming unit method. The TS agar was prepared by dissolving 30 g of TS broth in double-distilled water. Then, 30 g of TS agar was added, autoclaved (at 120 ºC for 15 min at 15 psi.) and cooled down to 50 ºC by keeping at room temperature. No antibiotics were used during experiments. The solution was poured into plates and allowed to cool down to room temperature under aseptic conditions. The plates were (inverted) stored at 4 °C in a refrigerator and pre-heated for 1 h before use.

Section II: Material Preparation for Antifungal Activity and Anti-fungal Testing

To prepare the agar plates, the culture medium was prepared by dissolving 3 g of ammonium nitrate, 2.5 g of potassium dihydrogen phosphate, 2 g of dipotassium hydrogen phosphate, 0.2 g of magnesium sulfate, 0.1 g of ferrous sulfate and 20 g of agar, dissolved to 1000 mL of double-distilled water. The culture was autoclaved (121 °C, 30 min, 15 psi) and cooled down to 50 °C by keeping at room temperature. The warm agar solution was filled in the petri dish and allowed to harden under aseptic conditions. For antifungal testing, a fresh piece of filter paper was steaked by the test fungi using a sterile needle and incubated in an oven at 30 °C for two weeks to ensure abundant growth. The filter paper was removed from the bottle, and the spores were suspended into double-distilled water by vigorous shaking. The drop-solution was used to infect the pre-coated agar plate at its middle position. The coating was created by drop casting 50 μL of various test solutions and spreading all over the plate with the help of an L-shaped disposable spreader. After infecting the plate with the test fungi, the plate was incubated at room temperature.

A macroscopic level of growth was observed, and the diameter of the ring was noted and compared with the plate containing the control sample. The method was in agreement with the AATC procedure.

Table S2 The total number of colonies in all experimental runs.

				Escherichia coli				
**Sample**	**R1**	**% CFU**	**R2**	**% CFU**	**R3**	**% CFU**	**Mean**	**STDEV**
Control	336	100.00	329	100.00	350	100.00	100	0.00
Cinnamon	221	65.77	219	66.57	211	60.29	64	3.42
Ag@CINN	96	28.57	107	32.52	119	34.00	32	2.81
Ag@CINN-Lim	70	20.83	60	18.24	66	18.86	19	1.36
				Staphylococcus aureus				
**Sample**	**R1**	**% Residual**	**R2**	**% Residual**	**R3**	**% Residual**	**Mean**	**STDEV**
Control	3175	100.00	3000	100.00	3350	100.00	100	0.00
Cinnamon	1090	34.33	1220	40.67	890	26.57	34	7.06
Ag@CINN	252	7.94	260	8.67	248	7.40	8	0.63
Ag@CINN-Lim	45	1.42	47	1.57	38	1.13	1	0.22

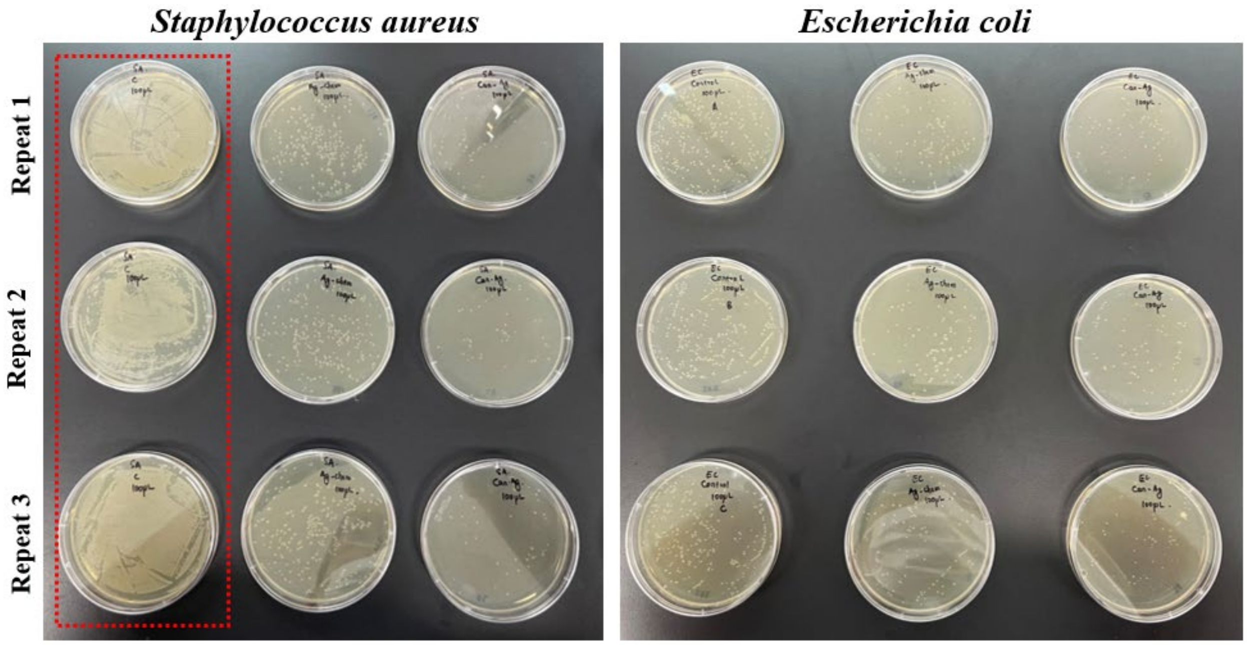

Figure S1. Photographic images of incubated agar plates for each of the three repetitions.

Section III: Estimated number of colonies in *S. aureus* (-tive control).

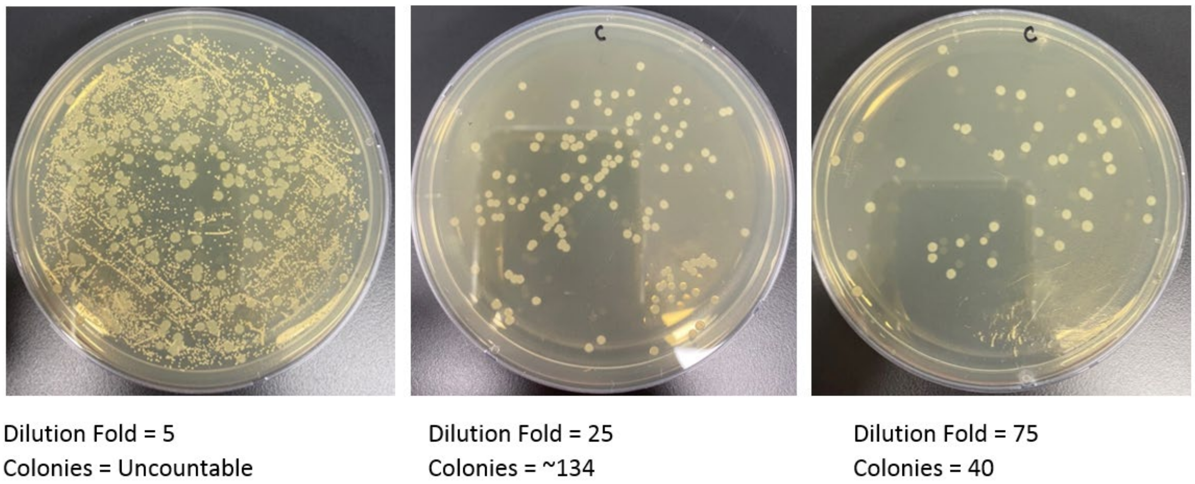

Figure S2. Estimated number of colonies in *S. aureus* (-tive control).

The PBS solution containing *S. aureus* was diluted to various dilution folds and inoculated on the TS agar plate without any poison (blank). The number of colonies was calculated, and according to the dilution folds, the actual number of colonies was estimated. Hence, we concluded that the number of colonies in the control sample was ~3175 (i.e., average of 25-DF and 75-DF).

Table S3 Fungal activity triplicate data for Ag@CINN and Ag@CIMM-Lim with controls.

	C. capsici				*F. oxysporum*			
	Diameter	Growth Percentage (%G)			Diameter	Growth Percentage (%G)		
	cm	**1st week**	**2nd week**	**Total**	cm	**1st week**	**2nd week**	**Total**
Blank	3.3			100	3.4			100
Positive Control	1.3	39.39		72.7	0.8	23.5		41.2
	2.4		33.31		1.4		17.7	
Ag@CINN	1	30.3		81.82	1.9	55.9		70.6
	2.7		51.52		2.4		14.7	
Ag@CINN-Lim	0.7	21.2		24.2	0.3	8.8		14.7
	0.8		3		0.5		5.9	

Abbreviations

Escherichia coli	*E. coli*
Staphylococcus aureus	*S. aureus*
Colletotrichum capsici	*C. capsici*
Fusarium oxysporum	*F. oxysporum*
